# Supplementary material for: The MYB-CC Transcription Factor PHOSPHATE STARVATION RESPONSE-LIKE 7 (PHL7) Functions in Phosphate Homeostasis and Affects Salt Stress Tolerance in Rice
Source: Plants (Basel). 2024 Feb 26;13(5):637. doi: 10.3390/plants13050637 (PMC10933727; doi:10.3390/plants13050637)
Supplement: Supplementary file 1 [file plants-13-00637-s001.zip › plants-2869902-supplementary.pdf]

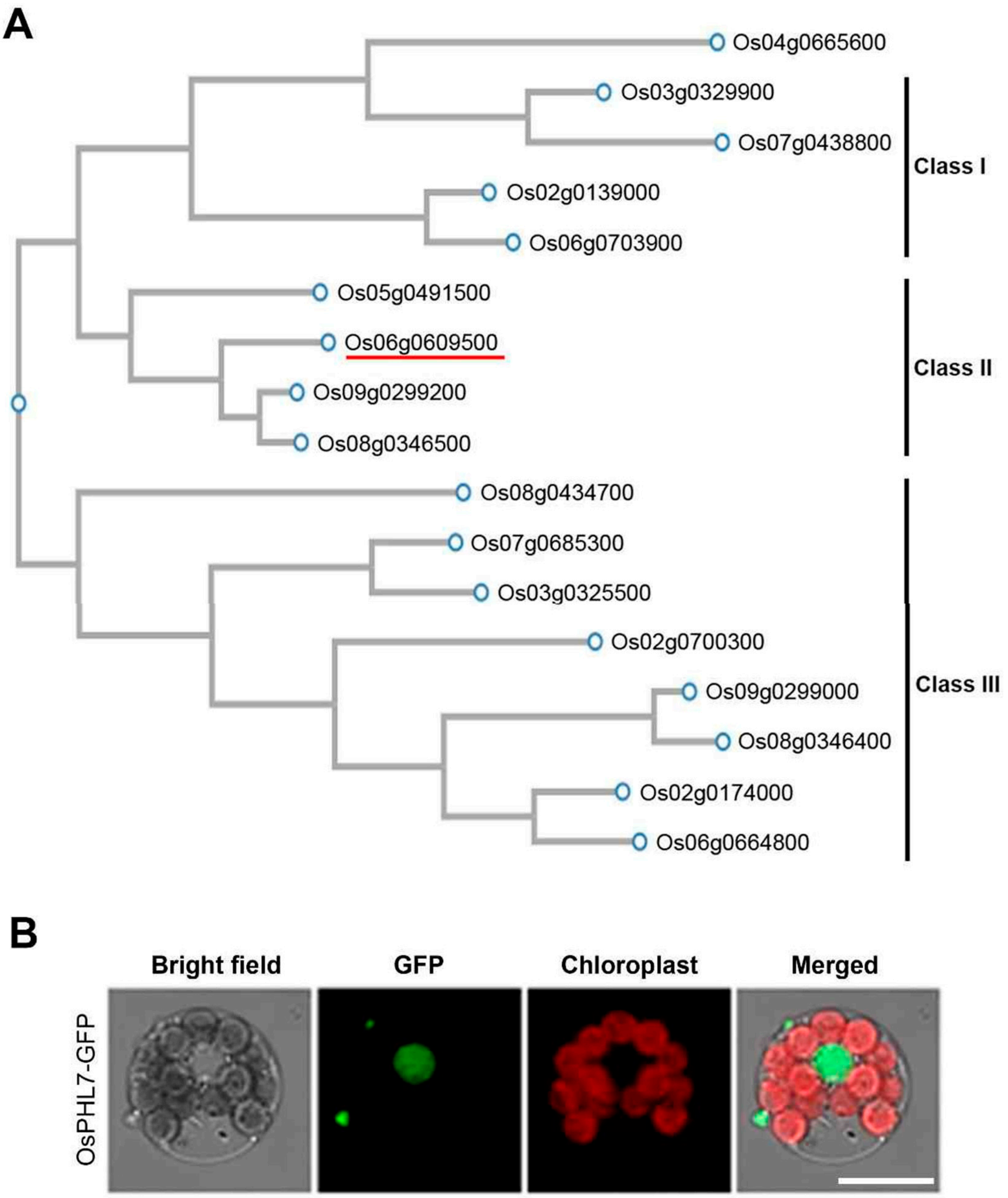

Figure S1. Subcellular localization and homology of OsPHL7. (**A**) Phylogenetic tree of the MYB family proteins related to OsPHL7 in rice. The red line indicates OsPHL7. Full-length amino acid sequences were used for these analyses. Protein accession numbers for the National Center for Biotechnology Information (www.ncbi.nlm.nih.gov) database are provided. (**B**) Transient expression of *OsPHL7-GFP* fusion constructs in protoplasts, indicating the subcellular localization of the resulting protein. Scale bars indicate 10 μm.

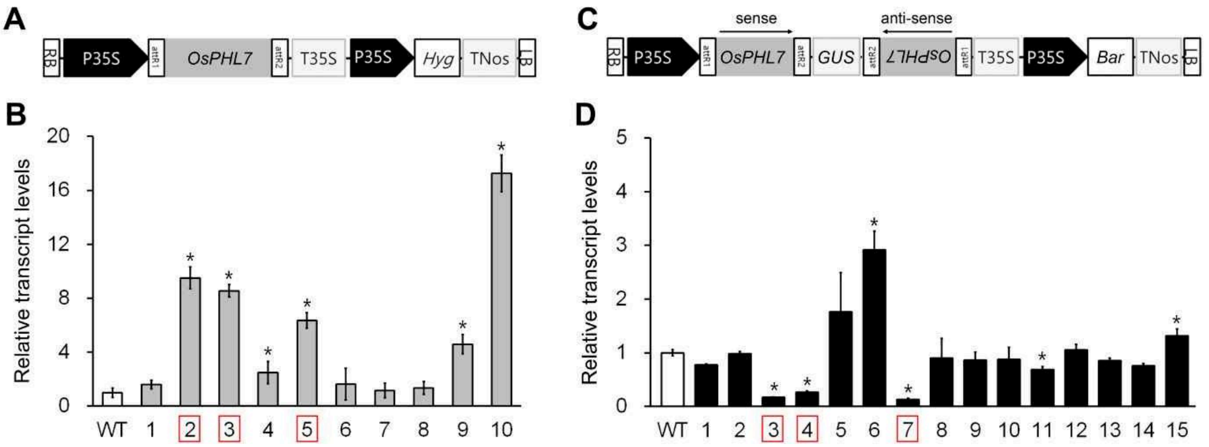

Figure S2. Analysis of *OsPHL7*-overexpressing and *osphl7*-RNA inference transgenic rice. (**A**) Schematic of the 35S::*OsPHL7* construct used to generate the *OsPHL7*- overexpressing (Ox) transgenic rice plants. (**B**) Expression levels of *OsPHL7* in *OsPHL7*-Ox. OsPHL7-Ox lines 2, 3, and 5 were selected for this study (red box). (**C**) A schematic of the *OsPHL7*-RNA interference (RNAi) construct used to generate the *osphl7*-knockdown mutants in rice. (**D**) Expression levels of *OsPHL7* in *osphl7*-RNAi. *osphl7*-RNAi lines 3, 4, and 7 were selected for this study (red box). Total RNA was extracted from 10-day-old transgenic plants of the T_3 generation, and the expression level was analyzed using qRT-PCR. The data represent mean values ± SD. Asterisks indicate statistically significant differences between the corresponding samples and their control ($p < 0.01$, 1-way ANOVA with Tukey post hoc test). *OsActin1* was used as the internal control, and the relative expression levels are shown as fold changes.

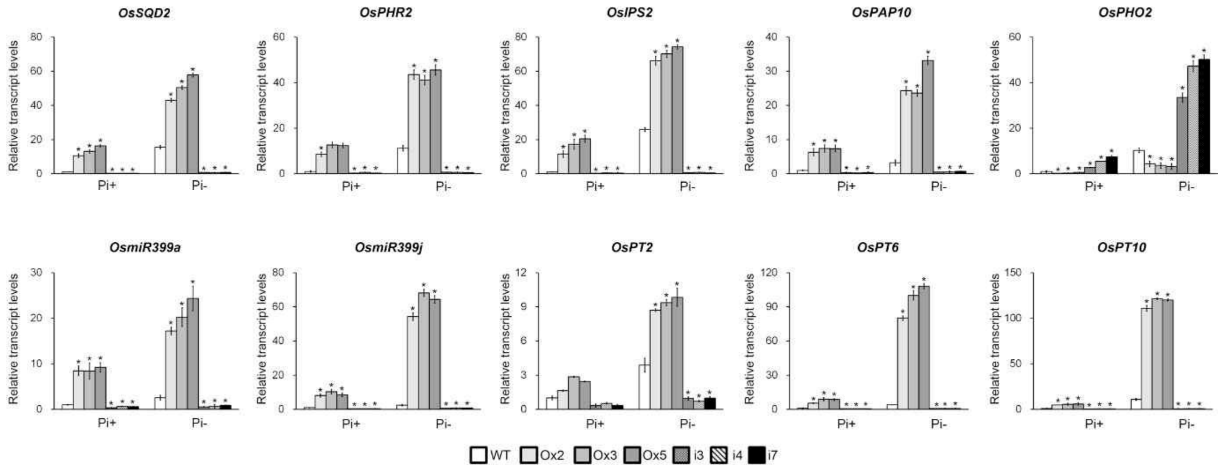

Figure S3. The expression patterns of Pi starvation response genes and Pi transport genes in the root of *OsPHL7*-overexpressing (Ox), and *osphl7*-RNA interference (RNAi) plants under Pi sufficient or deficiency conditions. RT-qPCR analysis of the expression levels of *OsSQD2, OsPHR2, OsIPS2, OsPAP10, OsPHO2, OsmiR399a, OSmiR399j, OsPT2, OsPT6,* and *OsPT10* in response to Pi-deficient conditions in the roots of 10-day-old *OsPHL7*-overexpressing (Ox), *osphl7*-RNA interference (RNAi), and wild-type (WT) plants. The plants were treated with 500 μM Pi (+Pi) or 20 μM Pi (−Pi) for 1 day. Ox2, Ox3, and Ox5 are three independent lines of P35S::*OsPHL7*; i3, i4, and i7 are three independent lines of *osphl7*-RNAi. The data are mean values of three biological replicates, and error bars indicate SD. Asterisks indicate statistically significant differences between the corresponding samples and their controls ($p < 0.01$, 1-way ANOVA with Tukey post hoc test). *OsActin1* was used as the internal control, and the relative expression levels are shown in fold values.

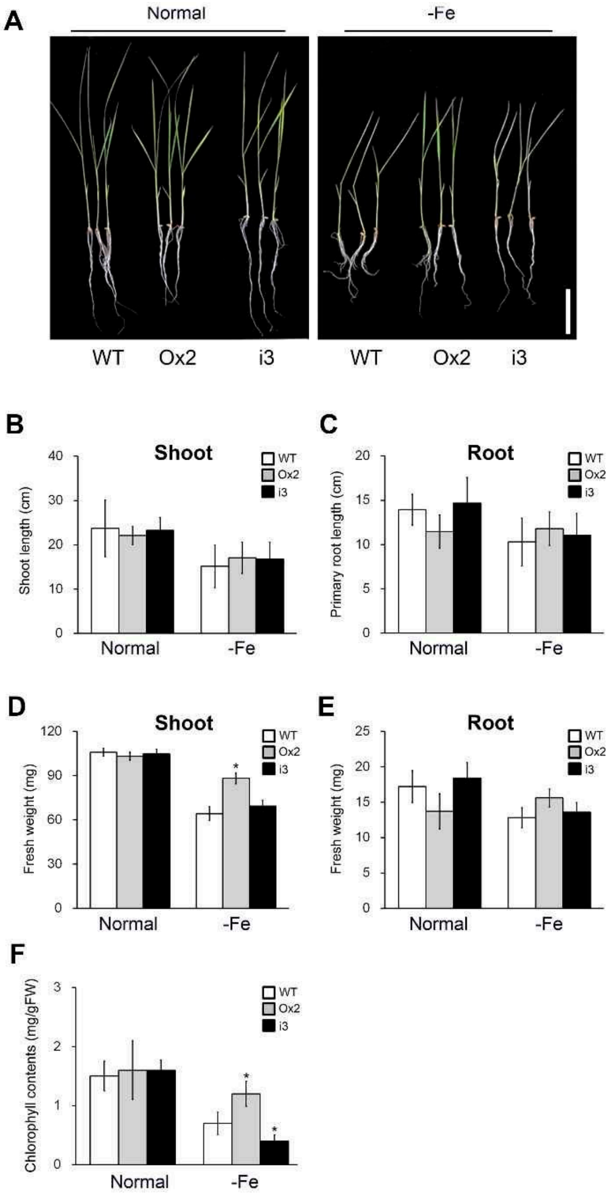

Figure S4. *OsPHL7*-overexpressing and *osphl7*-RNA interference plant responses to Fe deficiency. (**A**) The wild-type (WT), *OsPHL7*-overexpressing (Ox), and *osphl7*-RNA interference (RNAi) plants were grown for 10 days, after seedlings were transferred into Fe-sufficient and Fe-deficient conditions for 5 days. Scale bar indicates 5 cm. (**B**–**E**) Quantification of the lengths of the shoot (**B**) and primary root (**C**), and the fresh weights of the shoot (**D**) and roots (**E**) in their plants under both Fe-sufficient and Fe-deficient conditions. (**F**) Chlorophyll contents were measured in the shoots of plants under both treatment conditions. The data represent mean values ± SD (n = 30). Asterisks indicate statistically significant differences between the corresponding samples and their control ($p < 0.01$, 1-way ANOVA with Tukey post hoc test).

Table S1. Primer list in this study.

Gene	Direction	Sequence (5’→3’)	Purpose
OsPHL7	Forward	GTGCCAAGTCTGACAAGAAAG	Analysis of RT-qPCR
	Reverse	GCCTTTGCACCTCTAATTGTTC	
OsSQD2	Forward	CTGAAAACGGTAATGGATAGG	
	Reverse	AACAACAACAGCACGAGC	
OsPHR2	Forward	AGCCAATGCCTCAGTGAGAT	
	Reverse	GACCAGAATTGTCTGAAGGTTCTT	
OsIPS2	Forward	CTAAGGTAGGGCAACTTGTATC	
	Reverse	TTATTAGAGCAAGGACCGAAAC	
OsPAP10	Forward	ATACTGGCAGCCGACGGATGA	
	Reverse	GAGGGAGCTGGAGCGGAGAA	
OsPHO2	Forward	GGCTATCGGAACTTATGG	
	Reverse	AAGAAGGCAGAGGAGGTATC	
OsmiR399a	Forward	GCTGGAAATGATGCTGGTAGC	
	Reverse	CTCCTTTGGCACGAGATCTGT	
OsmiR399j	Forward	GGAGCATGTAAGTCTTTTGTAGC	
	Reverse	GGCAACTCTCCTTTGGCAGA	
OsPT2	Forward	GACGAGACCGCCCAAGAAG	
	Reverse	TTTTCAGTCACTCACGTCGAGAC	
OsPT6	Forward	CCGCCCCTGCAAACTGTA	
	Reverse	CAACTGGCGGTTTCTTCGAT	
OsPT10	Forward	ATGTCGCCCATCCTTCCA	
	Reverse	TCGCTTTCCGACGATGATC	
OsActin1	Forward	GAACTGGTATGGTCAAGGCTG	
	Reverse	ACACGGAGCTCGTTGTAGAAG	
OsPHL7	Forward	GGTGGGAACAGCTCAAACAG	For northern blot
	Reverse	GATTCACCCAGCACACCACT	
